# Supplementary material for: Antibiotic Resistance and Phylogeny of Pseudomonas spp. Isolated over Three Decades from Chicken Meat in the Norwegian Food Chain
Source: Microorganisms. 2021 Jan 20;9(2):207. doi: 10.3390/microorganisms9020207 (PMC7909287; doi:10.3390/microorganisms9020207)
Supplement: Supplementary file 1 [file microorganisms-09-00207-s001.pdf]

microorganisms

MDPI

Supplementary Materials: Antibiotic Resistance and Phylogeny of *Pseudomonas* spp. Isolated over Three Decades from Chicken Meat in the Norwegian Food Chain

Even Heir *, Birgitte Moen, Anette Wold Åsli, Marianne Sunde and Solveig Langsrud

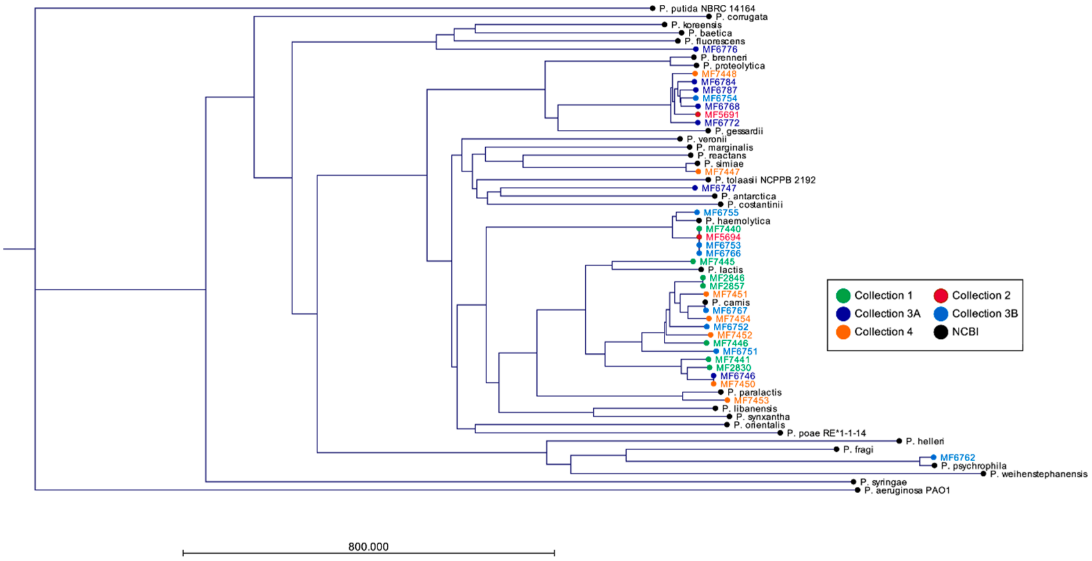

Figure S1. NDtree of the genome sequenced isolates (n = 31) together with 29 selected representative isolates from NCBI. The NCBI isolates are shown by species name in black and the 31 isolates from this study are colored according to collection number.

Citation: Heir, E.; Moen, B.; Åsli, A.W.; Sunde, M.; Langsrud, S. Antibiotic resistance and phylogeny of *Pseudomonas* spp. isolated over three decades from chicken meat in the Norwegian food chain *Microorganisms* **2021**, *9*, 207. https://doi.org/10.3390/microorganisms9020207

Received: 20 December 2020
Accepted: 18 January 2021
Published: 20 January 2021

Publisher's Note: MDPI stays neutral with regard to jurisdictional claims in published maps and institutional affiliations.

Copyright: © 2021 by the authors. Submitted for possible open access publication under the terms and conditions of the Creative Commons Attribution (CC BY) license (http://creativecommons.org/licenses/by/4.0/).

Table S1. Information and statistics on draft genome assembly data for 31 *Pseudomonas* spp. strains.

Assembly	# contigs (≥ 500 bp)	Total length (≥500 bp)	Largest contig	GC (%)	N50	CDS (prokka)	rRNA	Repeat region	Run No	Average coverage	Collection no.
MF2830	66	5974525	756123	60.21	225689	5430	4		2	90	1
MF2846	49	6201956	940246	60.14	243731	5580	6		2	82	1
MF2857	46	6201932	606173	60.14	333850	5581	6		2	87	1
MF5691	63	6222407	578174	60.42	240657	5598	6		1	356	2
MF5694	28	6042078	1062146	59.98	599652	5334	5		2	98	2
MF6746	98	6555296	410551	59.74	167740	5943	6		2	80	3A
MF6747	51	6811860	517135	60.05	297540	6156	6		2	74	3A
MF6751	105	6163290	397146	60.05	141886	5508	8		1	324	3B
MF6752	52	6100883	955559	60.09	322679	5450	8		2	95	3B
MF6753	36	6207278	1159705	59.64	367283	5536	6		2	102	3B
MF6754	76	6264719	612375	60.29	249574	5613	7		2	108	3B
MF6755	59	6561166	494347	59.49	265651	5849	5		1	282	3B
MF6762	77	5804763	410820	57.3	190055	5389	5		2	114	3B
MF6766	27	6379767	1749566	59.63	696963	5710	5		1	347	3B
MF6767	63	6547104	745903	59.74	256027	5937	7		2	77	3B
MF6768	95	6012416	426850	60.6	161035	5420	6		2	101	3A
MF6772	65	6218191	525726	60.69	237631	5643	6		2	96	3A
MF6776	117	6666116	466001	58.51	211334	6068	4		1	328	3A
MF6784	67	5970081	629089	60.67	266923	5370	8		2	96	3A
MF6787	62	6300351	1029761	60.28	237634	5682	7		1	230	3A
MF7440	36	6282835	829529	59.78	487444	5585	5		3	41	1
MF7441	68	6070436	627072	60.05	186108	5510	6		3	52	1
MF7445	101	6782664	572539	59.78	163205	6100	6		3	46	1
MF7446	44	6162814	738133	59.95	279499	5489	7		3	38	1
MF7447	55	6448003	748871	60.25	274573	5854	7		3	46	4
MF7448	84	6093941	796909	60.71	163092	5506	8		3	42	4
MF7450	131	6447082	337237	59.74	113604	5803	5		3	48	4
MF7451	53	6259359	467413	59.91	259080	5604	8		3	76	4
MF7452	73	6430424	452601	59.61	191638	5815	8		3	53	4
MF7453	55	6529967	767135	59.63	291860	5822	5	1	3	97	4
MF7454	78	6809055	555633	59.43	196228	6253	7		3	57	4

Table S2. List of 28 *Pseudomonas* genome assemblies downloaded from the NCBI microbial genome database and used in the NDtree analyses.

Assembly ID	Origin	Organism Name	Strain	Level
GCA_900113625.1	NCBI	*P. syringae*	BS3827	Contig
GCA_900105935.1	NCBI	*P. costantinii*	BS2773	Contig
GCA_900101415.1	NCBI	*P. koreensis*	BS3658	Chromosome
GCA_900101185.1	NCBI	*P. gessardii*	BS2982	Contig
GCA_013522805.1	NCBI	*P. carnis*	96A1	Contig
GCA_013385825.1	NCBI	*P. reactans*	C5002	Scaffold
GCA_012986545.1	NCBI	*P. lactis*	WS 5000	Contig
GCA_012986025.1	NCBI	*P. proteolytica*	WS 5126	Contig
GCA_011040435.1	NCBI	*P. psychrophila*	KM02	Complete
GCA_010448615.1	NCBI	*P. fluorescens*	DR397	Complete
GCA_009659615.1	NCBI	*P. haemolytica*	DSM 108988	Contig
GCA_004363635.1	NCBI	*P. brenneri*	BIGb0273	Scaffold
GCA_003851495.1	NCBI	*P. synxantha*	feb.79	Complete
GCA_002934065.1	NCBI	*P. orientalis*	F9	Complete
GCA_002813455.1	NCBI	*P. baetica*	LMG 25716	Contig
GCA_002813445.1	NCBI	*P. tolaasii* NCPPB 2192	NCPPB 2192	Contig
GCA_002269585.1	NCBI	*P. fragi*	F1786	Scaffold
GCA_002028325.1	NCBI	*P. veronii*	R02	Complete
GCA_001708425.1	NCBI	*P. corrugata*	RM1-1-4	Complete
GCA_001647715.1	NCBI	*P. antarctica*	PAMC 27494	Complete
GCA_001439735.1	NCBI	*P. paralactis*	DSM 29164	Contig
GCA_001439685.1	NCBI	*P. libanensis*	DSM 17149	Contig
GCA_001043065.1	NCBI	*P. helleri*	DSM 28141	Contig
GCA_001043055.1	NCBI	*P. weihenstephanensis*	DSM 29166	Contig
GCA_000967935.1	NCBI	*P. marginalis*	H21	Scaffold
GCA_000934565.1	NCBI	*P. simiae*	PCL1751	Complete

GCA_004412675.1	NCBI	*P. putida* NBRC 14164	NBRC 14164	Complete
GCA_000336465.1	NCBI	*P. poae* RE*1-1-14	RE*1-1-14	Complete
GCA_000006765.1	NCBI	*P. aeruginosa* PAO1	PAO1	Complete

Table S3. Species prevalence within the *Pseudomonas* strain collections 1, 2, 3A, 3B and 4. Best match species (partial 16S rRNA gene), number of isolates within that specie (n) and corresponding prevalence (%) within the different collections (1, 2, 3A, 3B and 4) are shown.

Best match (species)[1]	1	2	3A	3B	4	Grand total
P. gessardii (n = 68)	4%	9%	42%	11%	22%	21%
P. lactis (n = 57)	19%	9%	10%	39%	24%	18%
P. weihenstephanensis (n = 52)	35%	21%	8%	6%	0%	16%
P. canadensis (n = 22)	3%	3%	4%	33%	4%	7%
P. veronii (n = 22)	1%	9%	16%	0%	0%	7%
P. cerasi (n = 16)	7%	12%	4%	3%	0%	5%
P. corrugata (n = 13)	1%	9%	4%	3%	9%	4%
P. fragi (n = 13)	4%	6%	6%	0%	0%	4%
P. marginalis (n = 11)	4%	3%	1%	3%	9%	3%
P. migulae (n = 11)	2%	6%	5%	0%	2%	3%
P. helleri (n = 7)	6%	0%	0%	0%	0%	2%
P. koreensis (n = 6)	5%	3%	0%	0%	2%	2%
P. tolaasii (n = 6)	4%	3%	0%	3%	0%	2%
P. silesiensis (n = 5)	0%	0%	0%	0%	11%	2%
P. helmanticensis (n = 4)	2%	3%	0%	0%	2%	1%
P. turukhanskensis (n = 3)	1%	0%	0%	0%	4%	1%
P. deceptionensis (n = 2)	0%	3%	1%	0%	0%	1%
P. flavescens (n = 2)	0%	0%	0%	0%	4%	1%
P. brassicacearum (n = 1)	0%	0%	0%	0%	2%	0%
P. brenneri (n = 1)	0%	0%	1%	0%	0%	0%
P. caeni (n = 1)	0%	0%	0%	0%	2%	0%
P. rhodesiae (n = 1)	1%	0%	0%	0%	0%	0%
P. versuta (n = 1)	0%	3%	0%	0%	0%	0%

[1] Best match based on 288 bp og the 16S rRNA gene and the BLAST 16S rRNA (bacterial and archaea type strains) database.

Table S4. Taxonomy of isolates in Collection 1. The taxonomy based on carbon assimilation (Sundheim et al. 1998) and best BLAST match of partial 16S rRNA gene (288bp) are given for all isolates. The corresponding rMLST and nearest neighbour (NDtree) are given for the 7 isolates that were genome sequenced from Collection 1. Two isolates did not have a MF-number and are represented as na.

Strain	Carbon profile	Grouping according to	BLAST 16S best match	rMLST	Ndtree cluster

		**Sundheim et al. (1998)**			
MF2846	A	*P. fluorescens* biovar I-1	*P. lactis*	*Pseudomonas*	*P. lactis/ P. carnis*
MF7439	A	*P. fluorescens* biovar I-1	*P. lactis*		
MF7441	A	*P. fluorescens* biovar I-1	*P. lactis*	*P. fluorescens*	*P. lactis/ P. carnis*
MF2857	A	*P. fluorescens* biovar I-1	*P. lactis*	*Pseudomonas*	*P. lactis/ P. carnis*
MF7442	A	*P. fluorescens* biovar I-1	*P. lactis*		
MF2828	A	*P. fluorescens* biovar I-1	*P. lactis*		
MF2830	A	*P. fluorescens* biovar I-1	*P. lactis*	*P. fluorescens*	*P. lactis/ P. carnis*
na	A	*P. fluorescens* biovar I-1	*P. lactis*		
MF7445	A	*P. fluorescens* biovar I-1	*P. lactis*	*P. lactis*	*P. lactis/ P. carnis*
MF2815	A	*P. fluorescens* biovar I-1	*P. lactis*		
MF7547	A	*P. fluorescens* biovar I-1	*P. lactis*		
MF7446	A	*P. fluorescens* biovar I-1	*P. lactis*	*P. carnis*	*P. lactis/ P. carnis*
MF7553	A	*P. fluorescens* biovar I-1	*P. lactis*		
MF7490	B	very similar assimilation properties to group A strains	*P. canadensis*		
MF7440	B	very similar assimilation properties to group A strains	*P. canadensis*	*P. haemolytica*	*P. haemolytica*
MF7521	B	very similar assimilation properties to group A strains	*P. canadensis*		
MF7537	B	very similar assimilation properties to group A strains	*P. gessardii*		
MF7443	B	very similar assimilation properties to group A strains	*P. lactis*		
na	B	very similar assimilation	*P. lactis*		

		properties to group A strains	
MF7444	B	very similar assimilation properties to group A strains	*P. lactis*
MF7546	B	very similar assimilation properties to group A strains	*P. lactis*
MF7477	C	*P. lundensis*	*P. weihenstephanensis*
MF7484	C	*P. lundensis*	*P. weihenstephanensis*
MF7486	C	*P. lundensis*	*P. weihenstephanensis*
MF7500	C	*P. lundensis*	*P. weihenstephanensis*
MF7510	C	*P. lundensis*	*P. weihenstephanensis*
MF7512	C	*P. lundensis*	*P. weihenstephanensis*
MF7513	C	*P. lundensis*	*P. weihenstephanensis*
MF7523	C	*P. lundensis*	*P. weihenstephanensis*
MF7531	C	*P. lundensis*	*P. weihenstephanensis*
MF7543	C	*P. lundensis*	*P. weihenstephanensis*
MF7550	C	*P. lundensis*	*P. weihenstephanensis*
MF7551	C	*P. lundensis*	*P. weihenstephanensis*
MF7552	C	*P. lundensis*	*P. weihenstephanensis*
MF7519	D	Similar to group C	*P. koreensis*
MF7475	D	Similar to group C	*P. weihenstephanensis*
MF7476	D	Similar to group C	*P. weihenstephanensis*
MF7479	D	Similar to group C	*P. weihenstephanensis*
MF7481	D	Similar to group C	*P. weihenstephanensis*
MF7485	D	Similar to group C	*P. weihenstephanensis*
MF7497	D	Similar to group C	*P. weihenstephanensis*
MF7502	D	Similar to group C	*P. weihenstephanensis*

MF7506	D	Similar to group C	*P. weihenstephanensis*
MF7511	D	Similar to group C	*P. weihenstephanensis*
MF7504	D	Similar to group C	*P. weihenstephanensis*
MF7518	D	Similar to group C	*P. weihenstephanensis*
MF7527	D	Similar to group C	*P. weihenstephanensis*
MF7533	D	Similar to group C	*P. weihenstephanensis*
MF7549	D	Similar to group C	*P. weihenstephanensis*
MF7496	E1	*P. fluorescens* biovars IV-1 and V-1	*P. corrugata*
MF7501	E1	*P. fluorescens* biovars IV-1 and V-1	*P. migulae*
MF7503	E1	*P. fluorescens* biovars IV-1 and V-1	*P. turukhanskensis*
MF7555	E1	*P. fluorescens* biovars IV-1 and V-1	*P. weihenstephanensis*
MF7494	E2	E strains fell in between E1 og F	*P. helmanticensis*
MF7493	E2	E strains fell in between E1 og F	*P. koreensis*
MF7499	E2	E strains fell in between E1 og F	*P. koreensis*
MF7517	E2	E strains fell in between E1 og F	*P. koreensis*
MF7492	F	*P. fluorescens* biovar V-2	*P. helmanticensis*
MF7491	F	*P. fluorescens* biovar V-2	*P. koreensis*
MF2331	G	*P. fragi*	*P. cerasi*
MF7539	G	*P. fragi*	*P. cerasi*
MF7532	G	*P. fragi*	*P. fragi*
MF7480	G	*P. fragi*	*P. marginalis*
MF7509	H	Similar to group G	*P. cerasi*
MF7514	H	Similar to group G	*P. cerasi*
MF7534	H	Similar to group G	*P. cerasi*
MF7529	H	Similar to group G	*P. cerasi*
MF7530	H	Similar to group G	*P. cerasi*
MF7542	H	Similar to group G	*P. fragi*
MF7538	H	Similar to group G	*P. fragi*
MF7548	H	Similar to group G	*P. fragi*

MF7528	H	Similar to group G	*P. weihenstephanensis*
MF7482	I	closest similarity was with *P. fluorescens* biovar V-4	*P. helleri*
MF7526	I	closest similarity was with *P. fluorescens* biovar V-4	*P. helleri*
MF7535	I	closest similarity was with *P. fluorescens* biovar V-4	*P. helleri*
MF7541	I	closest similarity was with *P. fluorescens* biovar V-4	*P. helleri*
MF7554	I	closest similarity was with *P. fluorescens* biovar V-4	*P. helleri*
MF7558	I	closest similarity was with *P. fluorescens* biovar V-4	*P. helleri*
MF7524	J	*P. fluorescens* biovar II strains and biovar II-3	*P. marginalis*
MF2868	J	*P. fluorescens* biovar II strains and biovar II-3	*P. marginalis*
MF7544	J	*P. fluorescens* biovar II strains and biovar II-3	*P. rhodesiae*
MF7536	J	*P. fluorescens* biovar II strains and biovar II-3	*P. weihenstephanensis*
MF7438	K	*P. fluorescens* biovar II-1	*P. lactis*
MF7520	K	*P. fluorescens* biovar II-1	*P. marginalis*
MF7474	K	*P. fluorescens* biovar II-1	*P. tolaasii*
MF7483	K	*P. fluorescens* biovar II-1	*P. tolaasii*
MF7489	K	*P. fluorescens* biovar II-1	*P. tolaasii*
MF7498	K	*P. fluorescens* biovar II-1	*P. tolaasii*
MF7540	M	*P. fluorescens* biovar III-1	*P. gessardii*

MF7556	M	*P. fluorescens* biovar III-1	*P. gessardii*
MF7495	M	*P. fluorescens* biovar III-1	*P. veronii*
MF7516	N	not assigned	*P. gessardii*
MF7478	O	not assigned	*P. migulae*
MF7508	O	not assigned	*P. weihenstephanensis*
MF7487	P	not assigned	*P. weihenstephanensis*
MF7515	P	not assigned	*P. weihenstephanensis*
MF7545	P	not assigned	*P. weihenstephanensis*

Table S5. Antibiotic susceptibility data and presence/absence of class 1 integrons in 175 Pseudomonas spp. isolates.

				Antibiotics and MIC-values (mg/L)										
**Isolate (MF number)**	**Identification (BLAST 16S rDNA)**	**Isolate collection**	***intI*1 (class 1 integron)**	**Meropenem**	**Amikacin**	**Gentamicin**	**Aztreonam**	**Ciprofloxacin**	**Piperacillin/Tazobactam**	**Ceftolozane/ Tazobactam**	**Colistin**	**Tobramycin**	**Ceftazidime**	**Ceftazidime/Avibactam**	**Imipenem**
2331	*P. cerasi*	1	Neg	≤ 0.12	≤ 4	≤ 0.5	8	≤ 0.06	2/4	2/4	4	≤ 1	2	2/4	2
2830	*P. lactis*	1	Neg	16	≤ 4	≤ 0.5	64	≤ 0.06	4/4	1/4	≥ 16	2	2	2/4	16
2846	*P. lactis*	1	Neg	8	≤ 4	≤ 0.5	64	0.12	2/4	1/4	≥ 16	2	2	2/4	16
2857	*P. lactis*	1	Neg	8	≤ 4	≤ 0.5	64	0.12	4/4	1/4	≥ 16	2	2	2/4	16
2868	*P. marginalis*	1	Neg	8	≤ 4	≤ 0.5	64	0.25	8/4	2/4	8	≤ 1	8	8/4	4
5677	*P. migulae*	2	Neg	0.5	≤ 4	≤ 0.5	64	0.12	4/4	≤ 0.5/4	0.5	2	4	4/4	≤ 0.5
5678	*P. marginalis*	2	Neg	2	≤ 4	≤ 0.5	64	≤ 0.06	8/4	2/4	4	2	4	4/4	2
5680	*P. veronii*	2	Neg	2	≤ 4	≤ 0.5	64	≤ 0.06	16/4	2/4	≤ 0.25	2	8	4/4	1
5687	*P. tolaasii*	2	Neg	4	≤ 4	≤ 0.5	64	≤ 0.06	4/4	1/4	1	2	4	2/4	8
5688	*P. migulae*	2	Neg	0.5	≤ 4	≤ 0.5	32	≤ 0.06	8/4	≤ 0.5/4	1	2	2	2/4	1
5691	*P. gessardii*	2	Neg	32	≤ 4	≤ 0.5	32	≤ 0.06	8/4	1/4	1	2	16	4/4	16
5692	*P. veronii*	2	Neg	1	≤ 4	≤ 0.5	64	≤ 0.06	4/4	1/4	8	2	2	2/4	2
5694	*P. canadensis*	2	Neg	16	≤ 4	≤ 0.5	64	0.25	8/4	1/4	≥ 16	2	4	4/4	16

5696	*P. gessardii*	2	Neg	4	≤ 4	≤ 0.5	64	≤ 0.06	8/4	1/4	1	2	8	2/4	4
5697	*P. weihenstephanensis*	2	Neg	4	≤ 4	≤ 0.5	64	0.12	4/4	1/4	2	2	2	2/4	2
5701	*P. deceptionensis*	2	Neg	≤ 0.12	≤ 4	≤ 0.5	8	≤ 0.06	2/4	1/4	1	2	1	1/4	1
5702	*P. fragi*	2	Neg	≤ 0.12	≤ 4	≤ 0.5	16	≤ 0.06	4/4	2/4	1	2	4	4/4	≤ 0.5
5703	*P. corrugata*	2	Neg	0.25	≤ 4	≤ 0.5	32	≤ 0.06	8/4	≤ 0.5/4	0.5	2	4	4/4	≤ 0.5
5706	*P. cerasi*	2	Neg	≤ 0.12	≤ 4	≤ 0.5	16	≤ 0.06	4/4	2/4	4	2	4	4/4	2
5707	*P. helmanticensis*	2	Neg	0.5	≤ 4	≤ 0.5	32	0.12	8/4	≤ 0.5/4	1	2	2	2/4	1
5708	*P. koreensis*	2	Neg	0.25	≤ 4	≤ 0.5	64	≤ 0.06	16/4	1/4	1	2	≥ 32	4/4	2
5724	*P. lactis*	2	Neg	8	≤ 4	≤ 0.5	64	≤ 0.06	8/4	1/4	≥ 16	2	2	2/4	8
5729	*P. versuta*	2	Neg	≤ 0.12	≤ 4	≤ 0.5	1	≤ 0.06	≤ 1/4	≤ 0.5/4	8	2	≤0.5	≤ 0.5/4	≤ 0.5
5731	*P. weihenstephanensis*	2	Neg	≤ 0.12	≤ 4	≤ 0.5	8	0.12	4/4	1/4	1	2	1	1/4	1
6729	*P. corrugata*	3A	Neg	1	≤ 4	≤ 0.5	32	0.12	4/4	≤ 0.5/4	0.5	2	4	2/4	1
6730	*P. gessardii*	3A	Neg	8	≤ 4	≤ 0.5	64	≤ 0.06	8/4	1/4	0.5	2	8	4/4	4
6731	*P. lactis*	3A	Neg	2	≤ 4	≤ 0.5	64	≤ 0.06	2/4	≤ 0.5/4	≥ 16	2	2	1/4	2
6732	*P. cerasi*	3A	Neg	≤ 0.12	≤ 4	≤ 0.5	8	≤ 0.06	2/4	2/4	1	2	2	2/4	≤ 0.5
6734	*P. gessardii*	3A	Neg	4	≤ 4	≤ 0.5	64	≤ 0.06	8/4	2/4	1	2	4	4/4	2
6735	*P. weihenstephanensis*	3A	Neg	≤ 0.12	≤ 4	≤ 0.5	8	0.12	2/4	1/4	1	2	1	1/4	≤ 0.5
6736	*P. corrugata*	3A	Neg	≤ 0.12	≤ 4	≤ 0.5	32	≤ 0.06	4/4	≤ 0.5/4	1	2	2	2/4	≤ 0.5
6737	*P. weihenstephanensis*	3A	Neg	≤ 0.12	≤ 4	≤ 0.5	8	≤ 0.06	2/4	2/4	0.5	2	2	2/4	1
6738	*P. fragi*	3A	Neg	≤ 0.12	≤ 4	≤ 0.5	8	≤ 0.06	4/4	1/4	2	2	2	2/4	≤ 0.5
6739	*P. gessardii*	3A	Neg	16	≤ 4	≤ 0.5	64	≤ 0.06	8/4	1/4	1	2	8	2/4	8

6740	*P. canadensis*	3A	Neg	8	≤ 4	≤ 0.5	64	0.25	8/4	1/4	≥ 16	2	4	4/4	8
6741	*P. veronii*	3A	Neg	2	≤ 4	≤ 0.5	64	0.12	8/4	2/4	0.5	2	8	4/4	2
6742	*P. weihenstephanensis*	3A	Neg	≤ 0.12	≤ 4	≤ 0.5	16	0.12	2/4	1/4	2	2	2	2/4	8
6743	*P. fragi*	3A	Neg	≤ 0.12	≤ 4	≤ 0.5	16	≤ 0.06	8/4	1/4	2	2	4	4/4	2
6744	*P. migulae*	3A	Neg	0.5	≤ 4	≤ 0.5	64	0.12	4/4	≤ 0.5/4	0.5	2	2	2/4	≤ 0.5
6745	*P. gessardii*	3A	Neg	4	≤ 4	≤ 0.5	64	≤ 0.06	8/4	1/4	1	2	16	2/4	4
6746	*P. lactis*	3A	Neg	16	≤ 4	≤ 0.5	64	0.12	8/4	1/4	8	2	8	2/4	16
6747	*P. canadensis*	3A	Neg	8	≤ 4	≤ 0.5	64	≤ 0.06	4/4	1/4	≥ 16	2	4	4/4	16
6748	*P. cerasi*	3A	Neg	≤ 0.12	≤ 4	≤ 0.5	16	≤ 0.06	4/4	1/4	2	2	4	4/4	2
6749	*P. tolaasii*	3B	Neg	4	≤ 4	≤ 0.5	64	0.5	8/4	1/4	1	2	8	4/4	4
6750	*P. lactis*	3A	Neg	8	≤ 4	≤ 0.5	64	≤ 0.06	8/4	1/4	8	2	8	4/4	8
6751	*P. lactis*	3B	Neg	32	≤ 4	≤ 0.5	64	0.5	8/4	2/4	≥ 16	2	8	4/4	≥ 32
6752	*P. lactis*	3B	Neg	16	≤ 4	≤ 0.5	64	0.5	4/4	2/4	≥ 16	2	4	4/4	16
6753	*P. canadensis*	3B	Neg	16	≤ 4	≤ 0.5	64	0.25	8/4	1/4	≥ 16	2	4	2/4	8
6754	*P. gessardii*	3B	Pos	16	≤ 4	≤ 0.5	64	0.12	8/4	1/4	1	2	8	4/4	8
6755	*P. canadensis*	3B	Neg	32	≤ 4	1	64	1	16/4	4/4	2	≤ 1	8	8/4	16
6756	*P. corrugata*	3B	Neg	0.5	≤ 4	≤ 0.5	64	≤ 0.06	16/4	≤ 0.5/4	0.5	2	16	4/4	1
6757	*P. gessardii*	3B	Neg	4	≤ 4	≤ 0.5	64	≤ 0.06	8/4	1/4	0.5	2	8	2/4	4
6758	*P. marginalis*	3B	Neg	2	≤ 4	≤ 0.5	64	0.12	4/4	2/4	≥ 16	2	4	4/4	4
6759	*P. cerasi*	3B	Neg	2	≤ 4	≤ 0.5	32	≤ 0.06	4/4	≤ 0.5/4	0.5	2	4	4/4	2
6760	*P. marginalis*	3A	Neg	0.5	≤ 4	≤ 0.5	64	≤ 0.06	4/4	1/4	1	2	8	2/4	2
6762	*P. weihenstephanensis*	3B	Pos	0.25	≤ 4	≤ 0.5	8	2	4/4	2/4	1	2	2	2/4	1
6763	*P. gessardii*	3A	Neg	1	≤ 4	≤ 0.5	64	≤ 0.06	8/4	1/4	0.5	2	4	2/4	1

6765	*P. weihenstephanensis*	3A	Neg	≤ 0.12	≤ 4	≤ 0.5	4	≤ 0.06	≤ 1/4	1/4	0.5	2	1	1/4	≤ 0.5
6766	*P. canadensis*	3B	Neg	16	≤ 4	2	64	2	16/4	8/4	≥ 16	2	16	4/4	16
6767	*P. lactis*	3B	Neg	8	≤ 4	≤ 0.5	64	0.25	4/4	1/4	≥ 16	2	2	2/4	16
6768	*P. gessardii*	3A	Neg	16	≤ 4	≤ 0.5	64	≤ 0.06	8/4	1/4	1	2	8	2/4	16
6769	*P. deceptionensis*	3A	Neg	≤ 0.12	≤ 4	≤ 0.5	8	0.12	2/4	≤ 0.5/4	1	2	1	1/4	≤ 0.5
6770	*P. veronii*	3A	Neg	2	≤ 4	≤ 0.5	64	0.12	16/4	2/4	0.5	2	4	4/4	2
6771	*P. lactis*	3A	Neg	4	≤ 4	≤ 0.5	64	≤ 0.06	4/4	≤ 0.5/4	4	2	2	2/4	4
6772	*P. gessardii*	3A	Neg	32	≤ 4	1	32	≤ 0.06	8/4	1/4	1	2	≥ 32	2/4	16
6773	*P. migulae*	3A	Neg	1	≤ 4	≤ 0.5	64	≤ 0.06	2/4	≤ 0.5/4	0.5	2	≤ 0.5	≤ 0.5/4	1
6774	*P. canadensis*	3A	Neg	4	≤ 4	≤ 0.5	64	≤ 0.06	4/4	1/4	≥ 16	2	4	4/4	8
6775	*P. veronii*	3A	Neg	2	≤ 4	≤ 0.5	64	≤ 0.06	8/4	2/4	0.5	2	8	4/4	2
6776	*P. corrugata*	3A	Pos	32	≤ 4	≤ 0.5	64	0.25	32/4	≤ 0.5/4	1	2	16	≥ 16/4	4
6777	*P. migulae*	3A	Neg	0.5	≤ 4	≤ 0.5	64	≤ 0.06	4/4	≤ 0.5/4	0.5	2	2	2/4	1
6778	*P. veronii*	3A	Neg	2	≤ 4	≤ 0.5	64	0.12	8/4	2/4	0.5	2	8	4/4	2
6779	*P. weihenstephanensis*	3A	Neg	0.5	≤ 4	≤ 0.5	64	1	2/4	1/4	0.5	2	4	2/4	1
6780	*P. gessardii*	3A	Neg	4	≤ 4	≤ 0.5	32	≤ 0.06	4/4	≤ 0.5/4	0.5	2	4	1/4	4
6781	*P. lactis*	3A	Neg	2	≤ 4	≤ 0.5	64	≤ 0.06	4/4	1/4	≥ 16	2	2	2/4	2
6782	*P. weihenstephanensis*	3A	Neg	≤ 0.12	≤ 4	≤ 0.5	8	0.12	4/4	1/4	1	2	2	2/4	1
6783	*P. weihenstephanensis*	3A	Neg	≤ 0.12	≤ 4	≤ 0.5	8	0.12	2/4	1/4	1	2	1	1/4	≤ 0.5
6784	*P. gessardii*	3A	Neg	32	≤ 4	≤ 0.5	64	≤ 0.06	8/4	1/4	1	2	≥ 32	2/4	16

6785	*P. brenneri*	3A	Neg	4	≤ 4	≤ 0.5	64	≤ 0.06	8/4	1/4	1	2	2	2/4	1
6786	*P. veronii*	3A	Neg	2	≤ 4	≤ 0.5	64	≤ 0.06	8/4	1/4	≤ 0.25	2	4	4/4	2
6787	*P. gessardii*	3A	Neg	32	≤ 4	≤ 0.5	64	≤ 0.06	16/4	2/4	1	2	≥ 32	4/4	16
6788	*P. weihenstephanensis*	3A	Neg	≤ 0.12	≤ 4	≤ 0.5	16	≤ 0.06	4/4	1/4	2	2	2	2/4	2
6789	*P. lactis*	3A	Neg	4	≤ 4	≤ 0.5	32	0.12	4/4	2/4	8	2	2	2/4	8
6790	*P. fragi*	3A	Neg	≤ 0.12	≤ 4	≤ 0.5	8	≤ 0.06	4/4	≤ 0.5/4	1	2	2	2/4	≤ 0.5
6791	*P. fragi*	3A	Neg	≤ 0.12	≤ 4	≤ 0.5	8	≤ 0.06	2/4	≤ 0.5/4	0.5	2	2	2/4	≤ 0.5
6792	*P. fragi*	3A	Neg	≤ 0.12	≤ 4	≤ 0.5	16	≤ 0.06	4/4	4/4	2	2	8	4/4	≤ 0.5
6793	*P. cerasi*	3A	Neg	≤ 0.12	≤ 4	≤ 0.5	8	≤ 0.06	2/4	1/4	2	2	4	2/4	2
6794	*P. migulae*	3A	Neg	1	≤ 4	≤ 0.5	64	0.12	2/4	≤ 0.5/4	0.5	2	1	1/4	1
6795	*P. veronii*	3A	Neg	2	≤ 4	≤ 0.5	64	0.12	8/4	2/4	0.5	2	8	4/4	2
6808	*P. migulae*	3A	Neg	0.5	≤ 4	≤ 0.5	64	0.12	4/4	≤ 0.5/4	0.5	2	1	2/4	1
7440	*P. canadensis*	1	Neg	16	≤ 4	≤ 0.5	64	0.25	4/4	1/4	≥ 16	2	4	4/4	8
7441	*P. lactis*	1	Neg	4	≤ 4	≤ 0.5	64	≤ 0.06	4/4	1/4	≥ 16	2	2	2/4	16
7445	*P. lactis*	1	Neg	16	≤ 4	4	64	0.25	8/4	2/4	≥ 16	2	4	4/4	16
7446	*P. lactis*	1	Neg	8	≤ 4	≤ 0.5	64	0.25	4/4	2/4	≥ 16	2	4	2/4	16
7447	*P. marginalis*	4	Neg	4	≤ 4	≤ 0.5	64	0.12	8/4	2/4	4	2	≥ 32	4/4	8
7448	*P. gessardii*	4	Neg	16	≤ 4	≤ 0.5	64	≤ 0.06	8/4	2/4	1	2	16	4/4	16
7450	*P. lactis*	4	Neg	16	≤ 4	≤ 0.5	64	0.12	8/4	1/4	0.5	2	4	4/4	≥ 32
7451	*P. lactis*	4	Neg	16	≤ 4	≤ 0.5	64	0.25	4/4	2/4	≥ 16	2	4	4/4	16
7452	*P. lactis*	4	Neg	16	≤ 4	1	64	0.5	4/4	2/4	2	2	4	2/4	16
7453	*P. lactis*	4	Neg	8	≤ 4	≤ 0.5	64	0.25	4/4	2/4	4	2	8	4/4	16
7454	*P. lactis*	4	Neg	8	≤ 4	≤ 0.5	64	0.12	8/4	2/4	≥ 16	2	2	2/4	16
7478	*P. migulae*	1	Neg	1	≤ 4	≤ 0.5	64	0.12	4/4	≤ 0.5/4	0.5	2	2	2/4	1
7479	*P. weihenstephanensis*	1	Neg	0.25	≤ 4	≤ 0.5	16	≤ 0.06	4/4	1/4	1	2	2	1/4	1
7480	*P. marginalis*	4	Neg	0.5	≤ 4	≤ 0.5	64	≤ 0.06	8/4	1/4	1	2	4	1/4	1
7480	*P. marginalis*	1	Neg	2	≤ 4	≤ 0.5	64	≤ 0.06	4/4	1/4	≥ 16	2	2	2/4	4

7482	*P. helleri*	1	Neg	≤ 0.12	≤ 4	≤ 0.5	16	0.12	4/4	2/4	8	2	4	2/4	≤ 0.5
7483	*P. tolaasii*	1	Neg	4	≤ 4	≤ 0.5	64	≤ 0.06	8/4	1/4	1	2	8	4/4	4
7485	*P. weihenstephanensis*	1	Neg	0.5	≤ 4	≤ 0.5	16	0.12	4/4	2/4	1	2	2	2/4	1
7486	*P. weihenstephanensis*	1	Neg	≤ 0.12	≤ 4	≤ 0.5	16	0.12	4/4	2/4	1	2	4	4/4	1
7490	*P. canadensis*	1	Neg	4	≤ 4	≤ 0.5	64	0.25	8/4	1/4	≥ 16	2	8	4/4	8
7492	*P. helmanticensis*	1	Neg	1	≤ 4	≤ 0.5	64	0.12	16/4	1/4	1	2	4	4/4	≤ 0.5
7493	*P. koreensis*	1	Neg	0.25	≤ 4	≤ 0.5	64	0.12	8/4	1/4	0.5	2	≥ 32	4/4	2
7495	*P. veronii*	1	Neg	2	≤ 4	≤ 0.5	64	0.12	8/4	1/4	0.5	2	4	2/4	2
7496	*P. corrugata*	1	Neg	0.5	≤ 4	≤ 0.5	64	≤ 0.06	16/4	≤ 0.5/4	0.5	2	4	4/4	1
7497	*P. weihenstephanensis*	1	Neg	0.5	≤ 4	≤ 0.5	16	0.25	4/4	4/4	1	2	4	4/4	≤ 0.5
7498	*P. tolaasii*	1	Neg	4	≤ 4	≤ 0.5	64	≤ 0.06	8/4	1/4	1	2	4	4/4	4
7501	*P. migulae*	1	Neg	0.5	≤ 4	≤ 0.5	64	0.12	8/4	≤ 0.5/4	1	2	2	2/4	1
7503	*P. turukhanskensis*	1	Neg	0.5	≤ 4	≤ 0.5	64	≤ 0.06	8/4	1/4	0.5	2	≥ 32	4/4	1
7507	*P. silesiensis*	4	Neg	≤ 0.12	≤ 4	≤ 0.5	64	≤ 0.06	4/4	≤ 0.5/4	1	2	4	2/4	1
7508	*P. weihenstephanensis*	1	Neg	0.5	≤ 4	≤ 0.5	16	≤ 0.06	4/4	4/4	1	2	4	2/4	≤ 0.5
7509	*P. cerasi*	1	Neg	≤ 0.12	≤ 4	≤ 0.5	8	≤ 0.06	4/4	1/4	4	2	2	4/4	2
7510	*P. gessardii*	4	Neg	8	≤ 4	≤ 0.5	64	≤ 0.06	8/4	1/4	1	2	8	2/4	2
7512	*P. weihenstephanensis*	1	Neg	1	≤ 4	≤ 0.5	16	0.12	4/4	4/4	1	2	4	4/4	2
7514	*P. cerasi*	1	Neg	0.25	≤ 4	≤ 0.5	16	≤ 0.06	4/4	1/4	1	2	4	4/4	2

7515	*P. weihenstephanensis*	1	Neg	0.25	≤ 4	≤ 0.5	32	0.5	8/4	4/4	1	2	8	8/4	1
7516	*P. gessardii*	1	Neg	4	≤ 4	≤ 0.5	64	≤ 0.06	4/4	1/4	1	2	4	2/4	4
7517	*P. koreensis*	1	Neg	1	8	≤ 0.5	64	≤ 0.06	16/4	1/4	2	2	8	8/4	2
7518	*P. weihenstephanensis*	1	Neg	≤ 0.12	≤ 4	≤ 0.5	8	≤ 0.06	2/4	1/4	1	2	2	1/4	1
7519	*P. koreensis*	1	Neg	0.25	≤ 4	≤ 0.5	32	0.12	8/4	1/4	1	2	8	4/4	2
7520	*P. marginalis*	1	Neg	4	≤ 4	≤ 0.5	64	0.25	8/4	1/4	≥ 16	2	4	4/4	4
7521	*P. canadensis*	1	Neg	4	≤ 4	≤ 0.5	64	0.25	8/4	1/4	≥ 16	2	4	4/4	8
7524	*P. marginalis*	1	Neg	4	≤ 4	≤ 0.5	64	0.25	8/4	2/4	≥ 16	2	8	8/4	4
7525	*P. lactis*	4	Neg	4	≤ 4	≤ 0.5	64	0.12	4/4	1/4	4	2	2	2/4	8
7528	*P. weihenstephanensis*	1	Neg	≤ 0.12	≤ 4	≤ 0.5	16	0.12	4/4	4/4	4	2	4	4/4	≤ 0.5
7529	*P. cerasi*	1	Neg	≤ 0.12	≤ 4	≤ 0.5	8	≤ 0.06	4/4	≤ 0.5/4	2	2	2	2/4	0.5
7531	*P. silesiensis*	4	Neg	≤ 0.12	≤ 4	≤ 0.5	64	≤ 0.06	2/4	≤ 0.5/4	1	2	2	2/4	1
7531	*P. weihenstephanensis*	1	Neg	≤ 0.12	≤ 4	≤ 0.5	8	≤ 0.06	2/4	1/4	2	2	1	1/4	≤ 0.5
7532	*P. flavescens*	4	Neg	1	≤ 4	≤ 0.5	16	≤ 0.06	8/4	≤ 0.5/4	1	2	2	2/4	≤ 0.5
7532	*P. fragi*	1	Neg	≤ 0.12	≤ 4	≤ 0.5	16	≤ 0.06	2/4	1/4	1	2	4	4/4	≤ 0.5
7536	*P. flavescens*	4	Neg	2	≤ 4	≤ 0.5	16	≤ 0.06	8/4	≤ 0.5/4	1	2	2	2/4	≤ 0.5
7539	*P. cerasi*	1	Neg	≤ 0.12	≤ 4	≤ 0.5	8	≤ 0.06	2/4	1/4	4	2	2	2/4	2
7540	*P. gessardii*	1	Neg	8	≤ 4	≤ 0.5	32	≤ 0.06	4/4	1/4	1	2	4	2/4	8
7541	*P. marginalis*	4	Neg	4	≤ 4	≤ 0.5	64	0.12	4/4	≤ 0.5/4	8	2	2	2/4	4
7541	*P. helleri*	1	Neg	≤ 0.12	≤ 4	≤ 0.5	8	≤ 0.06	4/4	2/4	2	2	2	2/4	≤ 0.5

7543	*P. weihenstephanensis*	1	Neg	≤ 0.12	≤ 4	≤ 0.5	8	0.12	4/4	2/4	1	2	2	2/4	2
7544	*P. rhodesiae*	1	Neg	1	≤ 4	≤ 0.5	64	≤ 0.06	4/4	1/4	1	2	2	2/4	4
7548	*P. fragi*	1	Neg	0.12	≤ 4	≤ 0.5	16	0.06	4/4	2/4	8	2	4	4/4	2
7549	*P. weihenstephanensis*	1	Neg	0.25	≤ 4	≤ 0.5	16	0.12	4/4	2/4	1	2	2	2/4	1
7550	*P. weihenstephanensis*	1	Neg	≤ 0.12	≤ 4	≤ 0.5	8	0.12	2/4	1/4	1	2	2	1/4	≤ 0.5
7551	*P. weihenstephanensis*	1	Neg	0.5	≤ 4	≤ 0.5	8	0.12	4/4	2/4	2	2	1	1/4	1
7554	*P. helleri*	1	Neg	0.5	≤ 4	≤ 0.5	8	≤ 0.06	4/4	2/4	4	2	4	4/4	≤ 0.5
7556	*P. gessardii*	1	Neg	8	≤ 4	≤ 0.5	64	≤ 0.06	8/4	1/4	0.5	2	4	2/4	2
7557	*P. gessardii*	4	Neg	8	≤ 4	≤ 0.5	64	≤ 0.06	8/4	1/4	1	2	16	2/4	4
7558	*P. helleri*	1	Neg	0.25	≤ 4	≤ 0.5	16	0.12	4/4	4/4	4	2	8	4/4	≤ 0.5
7561	*P. corrugata*	4	Neg	0.5	≤ 4	≤ 0.5	32	≤ 0.06	8/4	≤ 0.5/4	0.5	2	4	2/4	≤ 0.5
7562	*P. gessardii*	4	Neg	8	≤ 4	≤ 0.5	64	0.12	8/4	1/4	0.5	2	8	4/4	2
7563	*P. koreensis*	4	Neg	1	≤ 4	≤ 0.5	32	≤ 0.06	4/4	1/4	0.5	2	8	4/4	2
7565	*P. gessardii*	4	Neg	2	≤ 4	≤ 0.5	32	≤ 0.06	4/4	1/4	0.5	2	4	2/4	1
7566	*P. silesiensis*	4	Neg	0.25	≤ 4	≤ 0.5	64	≤ 0.06	4/4	1/4	1	2	4	4/4	1
7567	*P. lactis*	4	Neg	4	≤ 4	≤ 0.5	64	≤ 0.06	4/4	1/4	≥ 16	2	2	2/4	4
7569	*P. gessardii*	4	Neg	4	≤ 4	≤ 0.5	64	0.12	8/4	1/4	4	2	4	4/4	2
7570	*P. turukhanskensis*	4	Neg	0.25	≤ 4	≤ 0.5	32	≤ 0.06	4/4	≤ 0.5/4	0.5	2	8	2/4	≤ 0.5
7571	*P. brassicacearum*	4	Neg	≤ 0.12	≤ 4	≤ 0.5	32	0.12	4/4	1/4	0.5	2	16	2/4	≤ 0.5

7572	*P. helmanticensis*	4	Neg	1	≤ 4	≤ 0.5	32	≤ 0.06	16/4	≤ 0.5/4	0.5	2	4	4/4	1
7573	*P. corrugata*	4	Neg	4	≤ 4	≤ 0.5	64	0.12	8/4	≤ 0.5/4	0.5	2	4	4/4	≤ 0.5
7574	*P. gessardii*	4	Neg	8	≤ 4	≤ 0.5	32	≤ 0.06	4/4	≤ 0.5/4	1	2	8	2/4	4
7575	*P. canadensis*	4	Neg	2	≤ 4	≤ 0.5	64	0.12	8/4	1/4	1	2	8	2/4	8
7576	*P. lactis*	4	Neg	4	≤ 4	≤ 0.5	64	0.12	4/4	1/4	≥ 16	2	2	2/4	8
7577	*P. lactis*	4	Neg	8	≤ 4	≤ 0.5	64	≤ 0.06	4/4	1/4	≥ 16	2	2	2/4	8
7578	*P. turukhanskensis*	4	Neg	2	≤ 4	≤ 0.5	64	≤ 0.06	16/4	1/4	1	2	≥ 32	4/4	1
7579	*P. migulae*	4	Neg	1	≤ 4	≤ 0.5	64	0.12	4/4	≤ 0.5/4	0.5	2	2	2/4	≤ 0.5
7580	*P. marginalis*	4	Neg	4	≤ 4	≤ 0.5	64	≤ 0.06	16/4	4/4	1	2	≥ 32	4/4	1
7582	*P. corrugata*	4	Neg	2	≤ 4	2	32	0.25	16/4	1/4	1	2	16	8/4	2
7584	*P. gessardii*	4	Neg	8	≤ 4	≤ 0.5	64	0.12	16/4	1/4	0.5	2	8	4/4	4
7585	*P. lactis*	4	Neg	4	≤ 4	≤ 0.5	64	0.12	4/4	1/4	≥ 16	2	4	2/4	8
7601	*P. gessardii*	4	Neg	8	≤ 4	≤ 0.5	64	≤ 0.06	8/4	1/4	0.5	2	4	2/4	4

Table S6. Taxonomic profiling based on ribosomal Multilocus Sequence Typing (rMLST). The isolate name, corresponding best match in the rMLST database and percentage support as well as best BLAST match according to partial 16S rRNA gene are show. For three isolates no species were given in rMLST, only 100% match to *Pseudomonas*.

Isolate	Best match	% support	BLAST 16S best match
MF6776	*P. baetica*	20	*P. corrugata*
MF5691	*P. brenneri*	97	*P. gessardii*
MF6768	*P. brenneri*	96	*P. gessardii*
MF6772	*P. brenneri*	98	*P. gessardii*
MF6784	*P. brenneri*	98	*P. gessardii*
MF6787	*P. brenneri*	98	*P. gessardii*
MF6754	*P. brenneri/P. proteolytica*	86/11	*P. gessardii*
MF7448	*P. brenneri/P. proteolytica*	89/6	*P. gessardii*
MF7446	*P. carnis*	100	*P. lactis*
MF7454	*P. carnis*	100	*P. lactis*
MF6751	*P. carnis/P. fluorescens*	50/50	*P. lactis*
MF2830	*P. fluorescens*	100	*P. lactis*
MF6746	*P. fluorescens*	100	*P. lactis*
MF6752	*P. fluorescens*	100	*P. lactis*
MF7441	*P. fluorescens*	100	*P. lactis*
MF7450	*P. fluorescens*	100	*P. lactis*
MF7451	*P. fluorescens*	98	*P. lactis*
MF7452	*P. fluorescens*	100	*P. lactis*
MF5694	*P. haemolytica*	100	*P. canadensis*
MF6753	*P. haemolytica*	100	*P. canadensis*
MF6766	*P. haemolytica*	100	*P. canadensis*
MF7440	*P. haemolytica*	100	*P. canadensis*
MF6755	*P. haemolytica/P. veronii*	88/4	*P. canadensis*
MF7445	*P. lactis*	100	*P. lactis*
MF7453	*P. paralactis/P. poae/P. synxantha*	87/5/5	*P. lactis*
MF6762	*P. psychrophila*	100	*P. weihenstephanensis*
MF7447	*P. simiae*	100	*P. marginalis*
MF6747	*P. veronii/**	25	*P. canadensis*
MF2846	*Pseudomonas*	100	*P. lactis*
MF2857	*Pseudomonas*	100	*P. lactis*
MF6767	*Pseudomonas*	100	*P. lactis*

* 12 % to *P. antarctica, P. costantinii, P. fluorescens, P. orientalis, P. reactans* and *P. tolaasii*
